# Transmission Potential of Chikungunya Virus and Control Measures: The Case of Italy

**DOI:** 10.1371/journal.pone.0018860

**Published:** 2011-05-03

**Authors:** Piero Poletti, Gianni Messeri, Marco Ajelli, Roberto Vallorani, Caterina Rizzo, Stefano Merler

**Affiliations:** 1 Bruno Kessler Foundation, Trento, Italy; 2 Institute of Biometeorology, National Research Council, Florence, Italy; 3 Consorzio LaMMa (Laboratory of Monitoring and Environmental Modelling for the sustainable development), Sesto Fiorentino, Italy; 4 National Research Council, Sesto Fiorentino, Italy; 5 National Center for Epidemiology Surveillance and Health Promotion, Istituto Superiore di Sanità, Rome, Italy; Massey University, New Zealand

## Abstract

During summer 2007 Italy has experienced an epidemic caused by Chikungunya virus – the first large outbreak documented in a temperate climate country – with approximately 161 laboratory confirmed cases concentrated in two bordering villages in North–Eastern Italy comprising 3,968 inhabitants. The seroprevalence was recently estimated to be 10.2%. In this work we provide estimates of the transmission potential of the virus and we assess the efficacy of the measures undertaken by public health authorities to control the epidemic spread. To such aim, we developed a model describing the temporal dynamics of the competent vector, known as *Aedes albopictus*, explicitly depending on climatic factors, coupled to an epidemic transmission model describing the spread of the epidemic in both humans and mosquitoes. The cumulative number of notified cases predicted by the model was 185 on average (95% CI 117–278), in good agreement with observed data. The probability of observing a major outbreak after the introduction of an infective human case was estimated to be in the range of 32%–76%. We found that the basic reproduction number was in the range of 1.8–6 but it could have been even larger, depending on the density of mosquitoes, which in turn depends on seasonal meteorological effects, besides other local abiotic factors. These results confirm the increasing risk of tropical vector–borne diseases in temperate climate countries, as a consequence of globalization. However, our results show that an epidemic can be controlled by performing a timely intervention, even if the transmission potential of Chikungunya virus is sensibly high.

## Introduction

During summer 2007 Italy has experienced the first large outbreak caused by Chikungunya virus (CHIKV) documented in a temperate climate country [1]. CHIKV is an arthropod–borne virus which can be transmitted to humans by *Aedes* mosquitoes [2], widespread in some tropical regions [3]–[8]. *Aedes albopictus* is highly competent for CHIKV [9], [10]. In Italy, the presence of this mosquito was first documented in Genoa and Padua (Northern Italy) in the earliest 1990's [11], [12]. Over the following years, *Aedes albopictus* expanded its distribution and is now well established in Northern and Central Italy [13], [14]. Having the potential to colonize the Mediterranean basin [15], the species has been reported from most Mediterranean European countries [16]. Samples of *Aedes albopictus* from the two villages were found to be positive for CHIKV sequences [1].

Sustained transmission of CHIKV was mainly observed in two neighboring villages in Emilia–Romagna region (North–Eastern Italy), namely Castiglione di Cervia and Castiglione di Ravenna [1], comprising 3,968 inhabitants in a built–up area of about 70 ha. The two villages are separated by a river with relatively stagnant water resulting from the presence of a lock. Houses are typically low (two storeys), surrounded by small gardens with many flowers, plants and flower pots. During the outbreak in the streets, drainage systems were visible, indicating open stagnant water underground [17].

A total of 161 laboratory confirmed cases were reported to the enhanced surveillance system developed in the two villages [1]. Sporadic cases, probably due to travel towards the most affected villages and not leading to sustained transmission, were also observed in other areas of the same region [1].

Moreover, a seroprevalence study, conducted on a random sample of residents in the village with the largest number of reported cases, shows a 10.2% of protected individuals [18]. Specifically, 82% were symptomatic – similar to 72.3% estimated in Mayotte, Indian Ocean [19] −85% of which satisfied the surveillance case definition, 63% of which were identified by the active surveillance system [18]. Higher prevalences were observed in La Reunion Island and in Mayotte, Indian Ocean, 38.2% and 37.2% respectively [19], [20].

The index case was recorded on June 23 2007 (a man who had arrived in Italy from India on June 21, [1]). On August 23, after the identification of CHIKV as the pathogen responsible for the ongoing epidemic, a set of interventions were undertaken to control the epidemic spread [1]: breeding sites and eggs removal on August 23; use of adulticides from August 23 to August 25 (3 days) and antilarval measures. Breeding sites were attempted to be removed in the entire area (house–to–house interventions were performed and community participation was encouraged as well) while insecticide interventions were undertaken within a radius of 100 m of each suspected case's residence (300 m for clusters of cases).

In this study we investigate the transmission potential of CHIKV in Italy, to provide insight into the possible impact of future outbreaks in temperate climate regions, and the effectiveness of the interventions performed during the outbreak, to provide insight into the epidemic control. To such aim, we developed a model describing the temporal dynamics of the competent vector, known as *Aedes albopictus*, explicitly depending on climatic factors, coupled to an epidemic transmission model describing the spread of the disease in both humans and mosquitoes, which allowed us to reproduce several observed features of the epidemic.

## Methods

**Figure 1:**
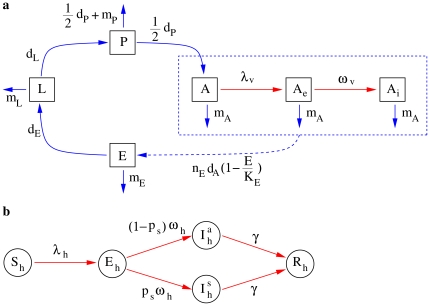
Schema of the model. **a** Model describing the evolution of the four life stages of the vector (eggs, 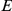, larvae, 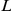, pupae, 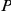, adult females 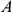; black boxes and blue arrows), coupled to the epidemic transmission model in vectors (susceptible, 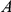, latent, 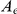, infectious, 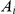; black boxes and red arrows). The dashed blue box and arrow refer to oviposition. **b** Model describing the epidemic transmission in humans (susceptible, 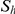, latent, 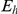, infectious symptomatic, 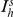, infectious asymptomatic, 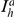, recovered, 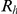; black circles and red arrows).

In Italy the competent vector for the transmission of CHIKV is *Aedes albopictus* [1]. CHIKV can spread from human to human through bites of adult female mosquitoes. As the dynamics of the vector depends, among several abiotic factors, on meteorological parameters, a population dynamics model accounting for seasonal temperature variations was used to estimate vector abundance. In particular, temperature plays a very significant role as it affects development and mortality rates of *Aedes albopictus* [10], [21], influencing vector abundance and distribution over time [22]. The population dynamics model was then coupled to an epidemic transmission model describing the spread of the epidemic in both humans and mosquitoes (see Fig. 1), allowing the estimate of the crucial parameters of the epidemic (e.g. basic reproduction number, effective reproduction number, probability that a major outbreak of the disease would occur after the introduction of a single infective host) and the assessment of intervention strategies.

### Modeling mosquito dynamics

The main purpose for modeling the dynamics of the vector is to give an approximate estimation of the abundance of female adult mosquitoes during the CHIKV epidemic outbreak in order to get a reasonable value of the ratio of mosquitoes to humans over time, a crucial factor for the calculation of the fundamental parameters of the epidemic and for the assessment of intervention measures. To achieve this goal, a differential equation model, structurally similar to those analyzed in [23]–[25], was introduced.

The dynamics of the mosquitoes over a land surface of about 70 ha (the extension of the study area) is described by a homogeneous mixing model. Briefly, the model simulates the abundance of the vector in the four life stages of *Aedes albopictus*, namely eggs (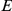), larvae (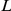), pupae (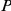), female adults (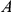), as follows:
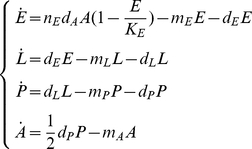
(1)where 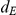, 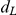, 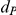 and 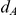 are the temperature dependent developmental rates; 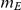, 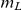, 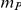 and 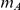 are the temperature dependent mortality rates; 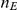 is the average number of eggs laid in one oviposition; 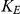 is the carrying capacity of eggs; the term 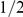 in the fourth equation accounts for the sex ratio (sex ratio is 1∶1, as reported in [21]). The four developmental rates correspond to egg hatching (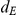), pupation (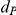), adult emergence (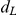) and gonotrophic cycle (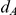). Length of the gonotrophic cycles subsequent to the first one and number of eggs laid at each gonotrophic cycles are not significantly different within a range of temperature between 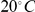 and 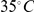 [21]. Therefore, we consider only one equation for modeling adults (and not a set of equations, describing transitions through gonotrophic cycles of different length, as in [23] for *Aedes Aegypti*) and the number of eggs laid in one oviposition does not depend on temperature.

### Modeling epidemic transmission

Human host population is assumed to be constant during the epidemic outbreak, given the brief duration of the epidemic compared to the lifespan of humans. We indicate with 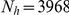 the number of humans [1]. As for the epidemic transmission model, hosts are classified as susceptible (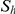), latent (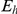), infectious symptomatic (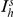) or asymptomatic (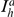), and recovered (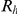). As only adult female mosquitoes are responsible for virus transmission, adult males are not explicitly represented in the transmission model. Female adult vectors are classified as susceptible (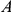), latent (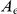) and infectious (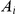). A susceptible vector enters the latent class after biting an infectious host at the per capita rate 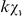, where 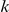 represents the biting rate of the vector (i.e., the number of bites to humans per mosquito per day) and 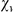 is the susceptibility to infection of the vectors (i.e., the probability that a mosquito get infected after biting an infectious host). A latent vector enters the infectious class after an average latent period of 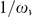 days and remains infectious for the rest of its life [26]. Thus, to account for the epidemic transmission process, the 4th Eq. of system (1) is replaced by the following three equations:
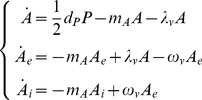
(2)where
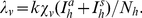
(3)Moreover, as we assume that the infection does not affect oviposition, the first Eq. of system (1) becomes:
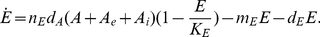
(4)


A susceptible host enters the latent class following the bite of an infectious vector at the per capita rate 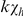, where 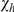 is the susceptibility to infection of humans. A latent host becomes infectious after an average latent period of 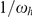 days, develops symptoms with probability 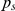 and then, after an average infectious period of 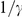 days, recovers.

The epidemic transmission process in humans can be modeled by the following system of ordinary differential equations, which has been added to system (1) as modified above (see Eq. 2 and 4):
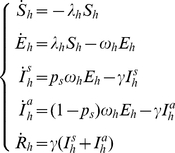
(5)where 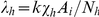. In what follows we refer to the full system coupling dynamics of the vector and epidemic transmission process as model 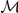.

### Basic reproduction number

The basic reproduction number 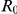 of host–vector infectious diseases is the number of secondary infections that arise when a single infective host is introduced into a fully susceptible host population through pathogen transmission by the vector [27]. The average number of hosts directly infected by the introduction of a single infective vector into a fully susceptible host population is given by the transmission probability 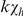 multiplied by the adult mosquito infectious lifespan (that is, the entire lifespan) 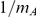:
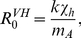
(6)The average number of vectors directly infected by the introduction of a single infective host into a fully susceptible vector population is given by the transmission probability 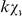 multiplied by the initial number of mosquitoes per human 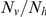 (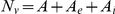 is the sum of all female adult mosquitoes, regardless of the epidemic status) that survive the latent period (probability: 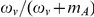), multiplied by the human infectious period 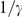:
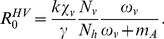
(7)Thus, the number of secondary infections generated by an infective host in a fully susceptible host population over the entire transmission cycle is:
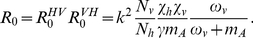
(8)Eq. (8), however, is the threshold parameter of a simplified model 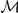 (with constant 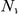). Therefore, to compute 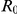 we assume a constant population of vectors, equal to the average value as predicted by the model in the initial phase of the epidemic, i.e. from June 21 to July 26 2007.

By employing the next–generation matrix method [28]–[30], one obtains the number of secondary cases generated either in hosts or vectors [31], that is the square root of Eq. (8).

Moreover, as shown in [32], the probability that a major outbreak of the disease would occur after the introduction of a single infective host is given by
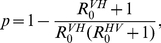
(9)where the terms 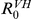 and 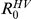 are defined in Eq. (6) and (7).

### Model parametrization

**Figure 2:**
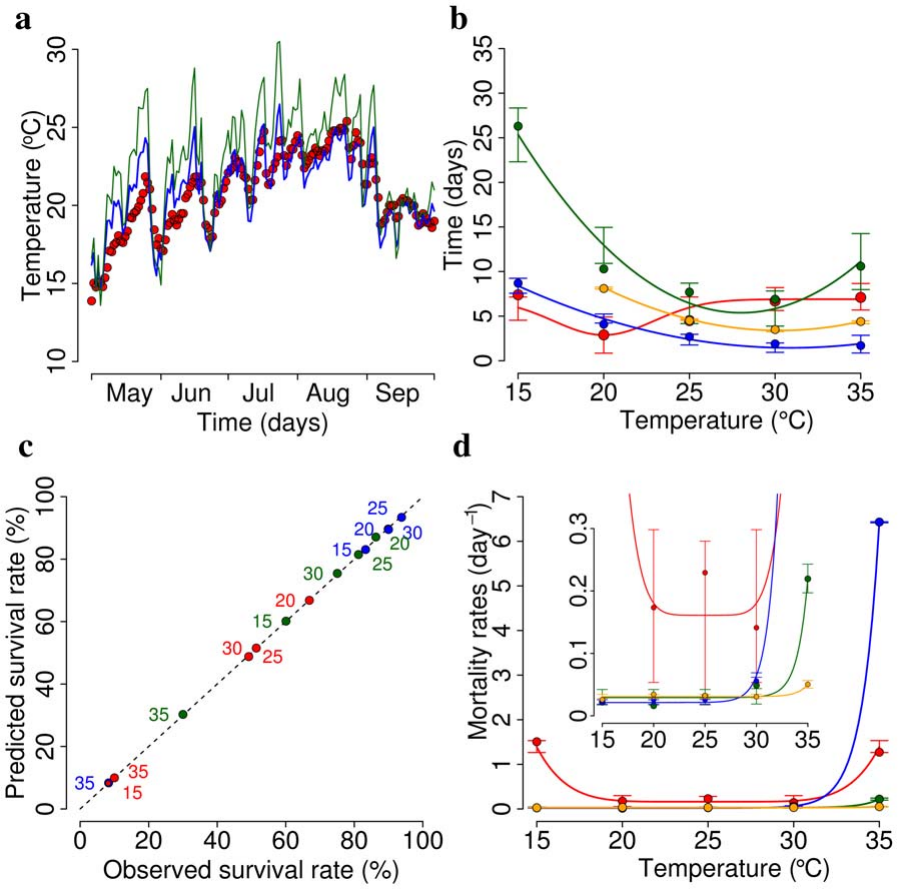
Model parametrization. **a** Observed average daily temperature inside a standard catch basin in 2009 (red points) versus values as predicted by a linear model whose independent variables are daily air temperature and relative humidity (blue line, RMSE = 1.3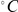) tuned on data from the 2008 season. Daily air temperature and relative humidity, measured two meters above the ground, were obtained from the urban weather station of Ravenna, the closest city to the two Italian villages affected by CHIKV. As for comparison, average daily air temperature is reported (green line), showing that water temperature is hardly comparable to air temperature (RMSE = 2.8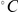). **b** Length of egg hatching period at temperatures 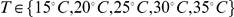, as reported in [21] (red circles), and predicted values (solid red lines); vertical bars represent 95% CI of predicted values. Other colors refer to length of larval development (green), pupal development (blue) and gonotrophic cycle (orange). **c** Survival rates of egg at temperatures 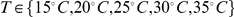, as reported in [21], versus predicted survival rates (red circles). Other colors refer to survival rates of larvae (green) and pupae (blue). **c** Mortality rates of eggs at temperatures 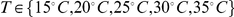, as derived by the analysis of survival rates (see Eq. 10, red circles), and predicted values (solid red lines); vertical bars represent 95% CI of predicted values. Other colors refer to mortality rates of larvae (green), pupae (blue) and adult females (orange).

On the basis of data presented in [21], we estimated the length of the developmental stages (egg hatching, larval and pupal development) and of the gonotrophic cycle as a function of temperature. To estimate, for instance, the length of the egg hatching period we used the following procedure: let 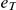 be the length of the egg hatching period for temperatures 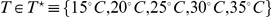, as reported in [21]. We assume that 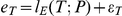 where 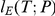 is a parametric function of the temperature 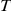 (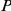 indicate the set of parameters) in a suitable set of functions, comprising exponential and parabolic functions, and 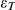 is a random sample of a 0 mean normal distribution with unknown variance 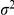. The square error 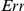 between predicted and observed length of the egg hatching period is defined as 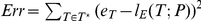. Parameters 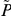 were estimated by minimizing 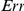. The variance 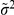 was computed as the average of the estimated residuals of the model (i.e., the average of the quadratic differences of 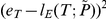 between the observed data and the best model fit 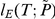). The uncertainty of the parameters was estimated by using a technique similar to that used in [33]. Specifically, we simulated 1000 different 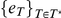, obtained by perturbing the best-fit 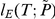 by adding a simulated error sampled from a normal distributed 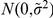 and we repeated the optimization procedure described above. Finally, the rate of eggs hatching is defined as 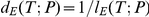. The same technique was used to estimate the length of larval and pupal development and the length of the gonotrophic cycle. Results are reported in Table 1. Fig. 2b shows a comparison between observed and modeled data.

**Table 1:** Length of the developmental stages.

development cycle	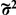	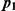 (95% CI)	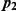 (95% CI)	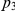 (95% CI)
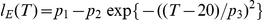	1.03	6.9 (5.7,9.9)	4.0 (1.8,7.2)	4.1 (0.2,17.2)
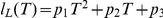	1.65	0.12 (0.08,0.15)	−6.6 (−8.3,−4.8)	98 (76.6,118.8)
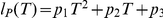	0.44	0.027 (0.018,0.038)	−1.7 (−2.2,−1.26)	27.7 (22.2,32.7)
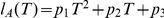	0.11	0.046 (0.038,0.049)	−2.77 (−2.96,−2.36)	45.3 (39.7,47.8)

We estimated mortality rates of eggs, larvae and pupae as a function of temperature on the basis of data on the survival rates presented in [21]. To estimate, for instance, the mortality rate of eggs we used the following procedure: let 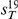 be the survival rates of eggs (19 days after oviposition) for temperatures 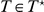, as reported in [21]. For a fixed value of temperature 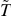, the following differential equation system describes the transition from eggs to larvae:
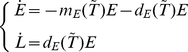
(10)where 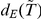 is the development rate as estimated above and 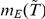 is the (unknown) mortality rate at the chosen temperature 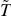. We chose 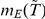 in such a way that, after 19 days, the survival rate as estimated through simulation of model (10) (i.e. the fraction of eggs that successfully develop into larvae) coincides with 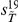. This procedure allowed the estimation of the mortality rate of eggs at temperatures 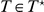. Fig. 2c shows that the estimated mortality rates of eggs at different temperatures lead to values of the survival rates compliant with the observed ones. The same procedure was used to estimate the mortality rates of larvae and pupae for temperatures 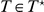. Mortality rates of adults for temperatures 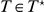 were directly available from [21]. The procedure described above for estimating the length of the developmental stages was used to estimate the mortality rates of all stages as a function of temperature in the range 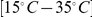. Results are reported in Table 2. Fig. 2d shows a comparison between observed and modeled data.

**Table 2:** Mortality rates.

stage	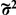	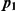 (95% CI)	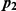 (95% CI)	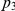 (95% CI)
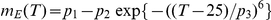	0.092	506 (43.4,925)	506 (43.4,925)	27.3 (18.3,30.8)
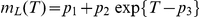	0.012	0.029 (0.017,0.041)	858 (20.6,973)	43.4 (39.7,43.5)
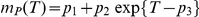	0.005	0.021 (0.018,0.026)	37 (14.8,57)	36.8 (35.8,37.2)
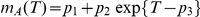	0.003	0.031 (0.028,0.04)	95820 (2954,98553)	50.4 (46.9,50.6)

Mortality rates as computed above depend only on temperature. Since parasitism and deficient nutrients have been found to cause a 35% increase in the rate of larval mortality [34] and adult *Aedes albopictus* females have been found to survive an average of only 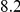 days (probability of daily survival = 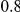 days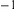) in the natural environment [35], mortality rates for immature stages (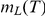 and 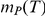) and adults (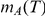) were multiplied by a factor 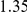 and 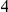 respectively.

The average number of eggs 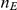 per oviposition is not significantly different at each gonotrophic cycle between 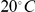 and 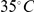 and in our simulations was uniformly chosen in the interval 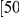 eggs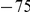 eggs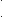, according to [21].

The carrying capacity of eggs 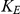 was estimated on the basis of data collected in the 2008 in the study area on the number of eggs per ovitrap per week as resulting from the analysis of 2741 ovitraps from week 21 to week 42 [36]. The mean egg density for the region of interest was found to be in the interval 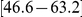 per ovitrap per week. Firstly, we estimated the carrying capacity of a single breeding site as the value 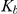 giving rise (through simulation of model (1), where all other parameters are known) to an estimated weekly incidence of eggs in the observed range (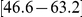) at temperatures observed in June and July. We estimated 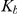 to be 19 in average (95% CI 14–27). The carrying capacity of the study region can be computed as 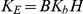, where 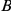 is the density of breeding sites (number per ha) and 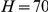 ha is the surface of the study area. The exact number of breeding sites (public and private catch basins, stagnant pools of water, etc.) in the area at the time of the epidemic is unknown. Hence, in this study we considered different values of 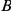, namely 50, 100, 150 and 200 ha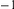, in order to describe different (high) densities of mosquitoes, as those observed in the study area [1]. These different scenarios are thus characterized by average values of of the carrying capacity 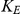 in the range 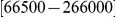.

As for the dependence of rates on temperature, the developmental rates of the aquatic stages, namely egg hatching, larval and pupal developments, and the mortality rates of eggs, pupae and larvae, are daily calculated as a function of the water mean temperature, while the length of the gonotrophic cycle and the mortality rate of adults are calculated as a function of air mean temperature. Since 2008 a monitoring activity has been carried out in order to estimate the water temperature (a key parameter in the developmental stages) of breeding sites. In fact, most of *Aedes albopictus* life stages develop in aquatic micro–environment. Specifically, a linear regression model was used to estimate the daily mean water temperature as a function of daily mean, maximum and minimum air temperature and daily mean air relative humidity (see Fig. 2a). This allowed us to get estimates of the water temperature for the 2007 season in order to get a more truthful calculation of developmental rates for eggs hatching and immature stages (larvae and pupae), impossible to obtain otherwise.

Plausible ranges for parameters most related to the epidemic transmission process were taken from literature (see Table 3), except for the biting rate 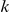 – it may vary a lot depending on human and mosquitoes populations, climatic and environmental factors [37]. We explored values of 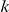 in the range 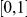 days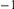.

**Table 3:** Epidemic parameters.

parameter	description	value	reference
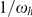	Latent period in humans	2–4 days	[3], [58]–[60]
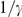	Infectious period in humans	2–7 days	[58], [59], [61]
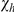	Human susceptibility to infections	50%–80%	[58]
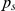	Symptomatic ratio	82%	[18]
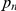	Notification ratio	54%	[18]
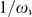	Latent period in mosquitoes	2–3 days	[26], [58]
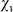	Mosquito susceptibility to infections	70%–100%	[62]

### Implementation and parameters optimization

To account for the stochastic nature of the processes regulating both dynamics of mosquito and epidemic transmission, we used a discrete–time stochastic version of model 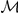, with time–step 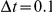 days. The time step was chosen short enough to obtain, on average, results comparable with those obtained by simulating the deterministic version of the model. Infections (either in vectors or hosts) occur with probability
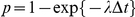
where 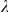 is the force of infection (see for instance [38]–[40]). For instance, to simulate the number of new infections in mosquitoes at a given time of the simulation, we sample from a binomial distribution with probability 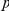, where 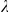 is 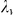 given in Eq. (3), and sample size 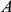, where 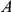 is the number of susceptible mosquitoes. Other transitions, e.g. through different life stages of the mosquito or through different epidemic classes, occur at the rate 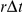 where 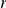 is the suitable rate (e.g. inverse of infectious period, inverse of the eggs hatching period, etc.).

All model parameters were calibrated to minimize the score function 
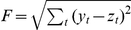
, where 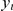 is the observed daily number of notified cases at time 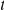 and 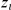 is the daily number of notified cases as predicted by the model at time 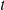 (times 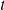 represent days before the intervention). Model 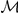 outputs the daily incidence of symptomatic cases, 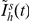, and asymptomatic cases, 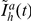. Notified cases 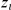 at time 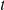 were estimated by sampling from a binomial distribution of size 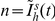 and probability 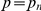, where 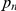 is the notification ratio (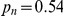 [18]).

Latin Hypercube Sampling (LHS) allows an efficient sampling of the parameter space which requires a smaller sample size than simple sampling to achieve the same accuracy [41]. LHS was used to build 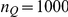 sets of parameters 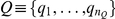 (uniform sampling was used to determine the 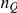 values of each model parameter) and thus, through model simulations, 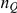 time series of predicted notified cases 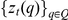. The optimal parameter set 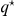 was chosen as the one minimizing the score function, i.e. 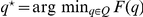. We repeated the above described procedure 100 times. This allowed us to estimate distributions of model parameters and, consequently, of the other quantities of interest (e.g. 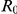, probability of major outbreak, attack rate). The index case was recorded on June 23 2007 (a man who had arrived in Italy from India on June 21, [1]) and thus we initialized all simulations with 1 infected individual on June 23. Results of the optimization procedure are shown in Fig. S1. Fig. S2 shows that 100 simulations are sufficient to obtain meaningful distributions of parameters and quantities of interest.

## Results and Discussion

**Figure 3:**
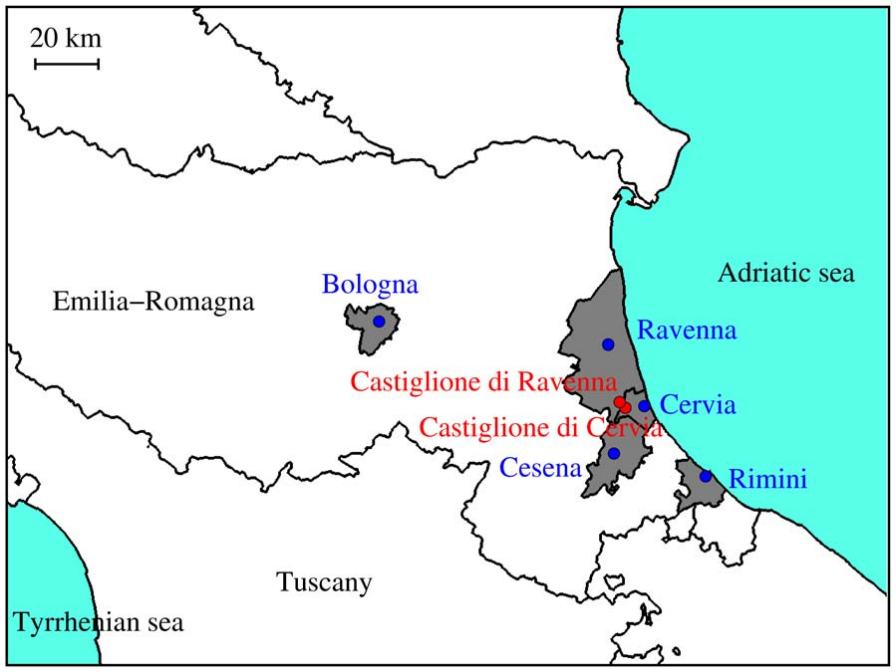
Study area. Geographical position of the two most affected villages (Castiglione di Cervia and Castiglione di Ravenna, in red), and municipalities where clusters of local transmission were observed (grey areas represent municipalities, blue points represent the geographic position of the main towns within municipalities).

In summer 2007, an outbreak of chikungunya fever affected the Italian provinces of Ravenna, Cesena-Forli, Rimini and Bologna [1], [42]–[44]. Health authorities identified 214 laboratory-confirmed cases with date of onset from July 15 to September 28 2007. Most cases (161) occurred in the two neighboring villages of Castiglione di Cervia and Castiglione di Ravenna, but five smaller clusters of local transmission were also detected in five towns in the same region (i.e., Cervia, Cesena, Ravenna, Rimini, and Bologna) which are located 9 to 75 km from the initially affected villages [1], [42]–[44], see Fig. 3.

**Figure 4:**
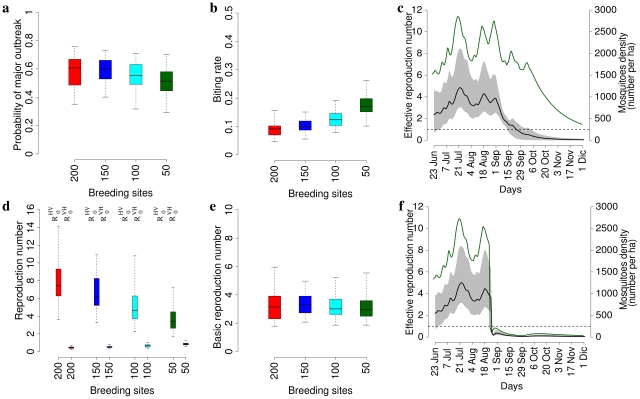
Potential transmission of CHIKV. **a** Distribution (2.5%, 25%, 50%, 75% and 97.5% percentiles) of the probability of observing a major outbreak for different numbers of breeding sites. **b** As **a** but for the biting rate. **c** Average effective reproduction number (black line, scale on the left) and 95% CI (grey area), and average density of mosquitoes (green line, scale on the right) over time by assuming 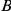 = 200 ha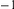 and no interventions. The dashed black line identifies the threshold value 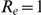. **d** As **a** but for 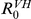 and 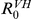 (see Eq. 6 and 7). **e** As **a** but for the basic reproduction number. **f** As **c** but by assuming reference interventions, resulting in the following reductions: 40% as for breeding sites and eggs, 90% as for larvae and 95% as for adults.

Model 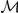 was parametrized to describe epidemic spread only in Castiglione di Cervia and Castiglione di Ravenna. Daily estimates of the number of vectors over time 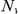 were obtained by the vector dynamics model. The ratio of mosquitoes to humans was estimated to be in the range of 10–35 during the peak mosquito activity (Fig. S3 show the predicted dynamics of the vector for different numbers of breeding sites 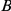). By fitting model 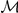 to notification data up to August 23 (the day before intervention) and by assuming 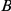 = 200 ha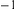 we estimated 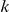 to be 0.09 days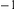 (95% CI 0.05–0.16 days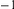, see Fig. 4b). We recall that the explored range for 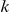 through the LHS procedure was 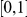. Good fit to data were obtained for values in the entire range explored for all the other model parameters (see Fig. S4).

The uncertainty of 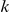 depends on the uncertainty of all model parameters. Unfortunately, no field data are available for the study area to validate these results. In [37], plausible values for the biting rate and the ratio of mosquitoes to humans in Europe are considered to be 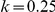 days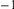 and 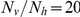 (based on published and non published data, e.g. [10]). However, we acknowledge that it might be misleading to compare these results with others carried out in other localities. In fact, abundance and biting rate of *Aedes Albopictus* are strongly affected by abiotic factors, both climatic and environmental (e.g. presence of other hosts).

Estimates of the biting rate and its uncertainty allowed us to estimate 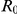 and its uncertainty from Eq. (8). Besides parameters more strictly related to the infectious process, 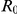 is an increasing function of the square of 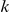, as the biting rate controls transmission from humans to mosquitoes and from mosquitoes to humans, and the ratio of vectors to humans 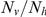 (see Fig. S5). However, it should be considered that Eq. (8) depends on the number of vectors 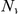 which substantially varies over time as a results of seasonal meteorological factors. Thus, as for models explicitly considering seasonal variations in transmission, it is difficult to precisely define 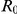. Therefore, we computed 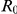 by considering the average value of 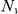 from June 21 to July 26 2007 (i.e. the initial phase of the epidemic), as predicted by the vector dynamics model. We estimated 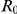 to be 3.3 on average (95% CI 1.8–6, see Fig. 4e). The estimated biting rate increases by decreasing the number of breeding sites and, consequently, estimates of 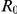 do not change substantially by varying the number of breeding sites (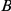 = 50 ha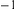: 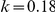 days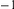, 95% CI 0.1–0.26 days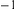, 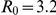, 95% CI 1.9–5.5; 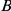 = 100 ha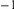: 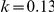 days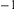, 95% CI 0.08–0.2 days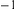, 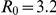, 95% CI 1.9–5.2; 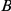 = 150 ha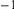: 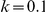 days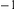, 95% CI 0.06–0.15 days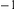, 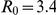, 95% CI 2.1–5, see Fig. 4b and Fig. 4e). Fig. 4d shows that 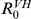 is below the critical threshold for all vales of 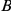 and thus the epidemic is mainly determined by 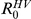, i.e. by transmission from humans to vectors. As for the effective reproduction number 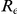 (i.e., the average number of secondary cases generated per primary case at a given time), which accounts for both depletion of susceptible individuals and mosquito dynamics, its value over time is shown in Fig. 4c and Fig. 4f (by considering or not interventions). It emerges that 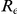, which does not change much by varying the number of breeding sites, can vary substantially over time as an effect of mosquito dynamics. This suggests that 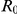 could have been even larger, depending on the time of epidemic seeding.

Recently, it has been demonstrated using mathematical modeling in the context of dengue that it is possible to generate outbreaks even in cases when 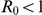 provided that the vector-to-human component of 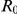 is greater than one and that a certain number of infected vectors are introduced into the affected population [45]. However, it has been demonstrated that the index case was a man of Indian origin from Kerala living in Castiglione di Cervia, without history of traveling during the previous year [1]. He only reported contact with a relative of his, who had arrived in Italy on June 21 2007 from Kerala, India (a region of India affected by the CHIKV epidemic), and visited him in Castiglione di Cervia village on June 23, while feverish. Therefore, having the human index case being identified, we can reasonably exclude the contemporaneous introduction of infected vectors in the two villages. Moreover, our estimates show that 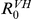 is well below the critical threshold.

As several cases were reported in Italy among travelers returning from endemic areas [46] (only one, however, in the study area; additional imported cases throughout the duration of the outbreaks were not detected), the question arises why no previous outbreaks of CHIKV occurred in other Italian regions. By assuming 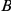 = 200 ha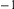, we estimated the probability 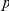 (see Eq. 9) that a major outbreak of the disease would occur after the introduction of a single infective host to be 0.59 (95% CI 0.35–0.76, see Fig. 4a). Estimates do not change substantially by varying the number of breeding sites (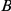 = 50 ha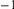: 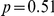, 95% CI 0.29–0.7, see Fig. 4a; 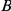 = 100 ha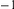: 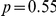, 95% CI 0.32–0.71, see Fig. 4a; 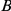 = 150 ha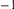: 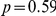, 95% CI 0.4–0.73, see Fig. 4a). For values of the biting rate 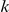 in a given range, results indicate that major outbreaks are possible only for large enough values of 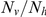 (see Fig. S5) and, by assuming the same density of mosquitoes, epidemic outbreaks are more likely in rural areas with respect to urban areas – as the human population density is much lower in the former. This could explain why cities like Cesena (96,000 inhabitants), Rimini (141,000 inhabitants) and Bologna (377,000 inhabitants) and Ravenna (157,000 inhabitants) located in the same region of the two most affected villages did not experience any epidemic outbreak, though sporadic CHIKV cases were recorded in the same period [1], [42]–[44]. These results support the hypothesis that outbreaks of Chikungunya virus in those temperate climate countries characterized by high density of *Aedes albopictus* are probable after the importation of an index case from abroad.

**Figure 5:**
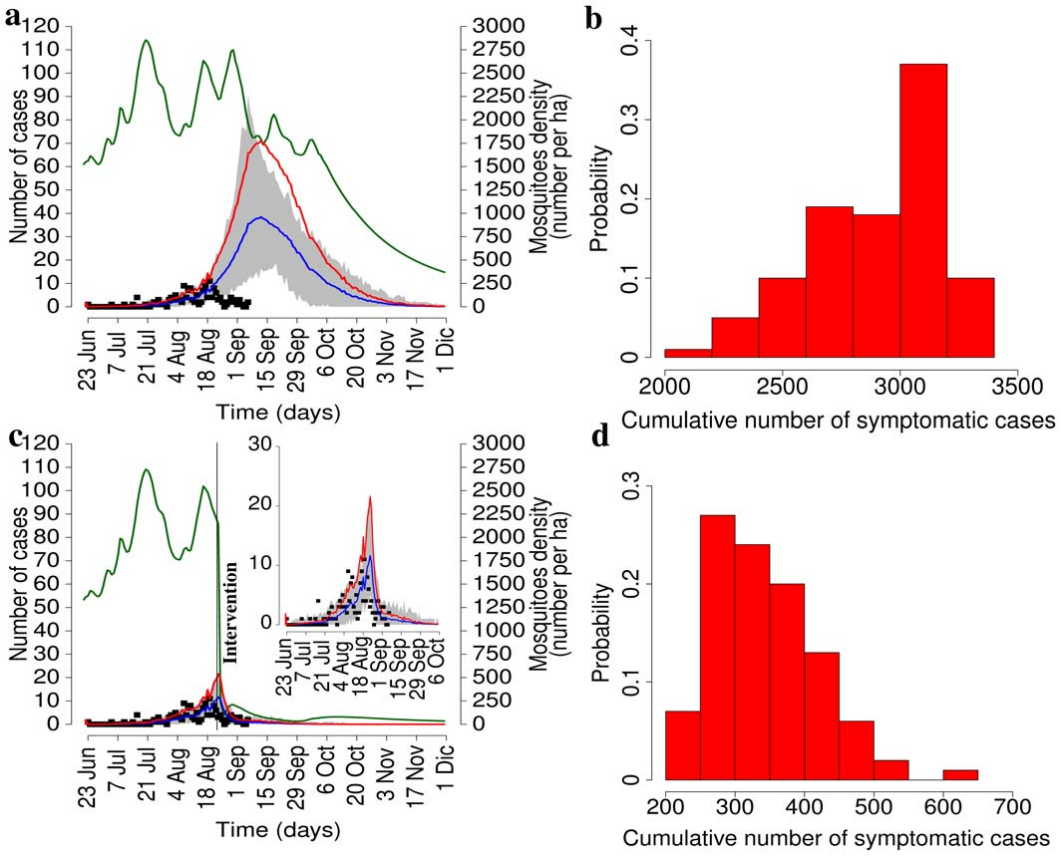
Baseline simulations and reference interventions. **a** Average daily number of symptomatic notified cases as predicted by the model in the absence of interventions (baseline scenario, blue line, scale on the left) and 95% CI (grey area) by assuming 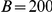 ha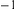, compared to the actual daily number of symptomatic notified cases (black points). Red line represents the overall average daily number of symptomatic cases as predicted by the model. Green line represents the average density of mosquitoes (scale on the right). **b** Histogram of the cumulative number of symptomatic cases as predicted by the model in the absence of interventions. **c** and **d** As **a** and **b** respectively but for assuming an intervention resulting in the following reductions: 40% as for breeding sites and eggs, 90% as for larvae and 95% as for adults (reference scenario).

The potential epidemic trajectory in the absence of interventions by assuming 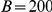 ha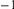 is shown in Fig. 5a. The resulting cumulative attack rate (i.e., the percentage of symptomatic cases in the population at the end of the epidemic) was estimated to be 73.4% of the population (95% CI 57.8%–81.5%, see also Fig. 5b). Results do not change much by varying 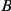 (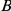 = 50 ha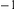: 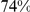, 95% CI 55.3%–81.6%, see also Fig. S6; 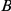 = 100 ha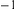: 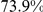, 95% CI 55.8%–81.5%; 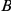 = 150 ha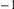: 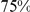, 95% CI 57.3%–81.3%). Much lower prevalence values have been estimated in La Reunion Island and in Mayotte, namely 38.2% and 37.2% respectively. However, these estimates are hardly comparable with our model predictions as these territories have benefited from high resource allocation to mitigate the epidemic [19].

As for the undertaken interventions, breeding sites and eggs were removed on August 23 2007, larvicides were used on August 23 (effect lasting 30 days), and adulticides were used from August 23 to August 25 2007. Through model simulations, we evaluated the effects of strategies mimicking the timing of the actual interventions undertaken in Italy. As for the effects in terms of reduction of breeding sites, eggs, larvae and adults, likely values are: 20% to 60% as for reduction breeding sites and eggs, 80% to 95% as for reduction of larvae and 80% to 95% as for reduction of adults. We discuss first results obtained by assuming 40% as for reduction of breeding sites and eggs, 90% as for reduction of larvae and 95% as for reduction of adults. The effects of such an intervention are shown in Fig. 5c. The resulting cumulative attack rate, by assuming 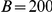 ha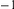, was estimated to be 8.7% (95% CI 5.6%–12.7%, see Fig. 5d), in good agreement with the observed value, namely 8.4%, computed by multiplying the overall observed prevalence, 10.2% [18], by the symptomatic ratio 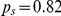 [18]. Results are similar for other choices of 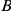 (for instance, for 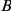 = 50 ha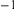 the figure becomes 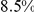, 95% CI 5.3%–13%; see also Fig. S6). To keep track of the number of symptomatic cases identified by the active surveillance system, we assume that human symptomatic cases are identified with probability 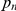 and this allows fitting notification data in model simulations. According to model estimates, the number of notified cases would have been about 185 on average (95% CI 117–278), in good agreement with the number as reported to the surveillance system, namely 161 cases [1]. The number of cases drastically decreased in late August while the effective reproduction number, in the absence of interventions, would have fallen below the epidemic threshold in late September (see Fig. 4c and Fig. 4f). This proves that a combined strategy resulted in a drastic reduction of the epidemic impact, despite the relatively large value of 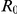.

**Figure 6:**
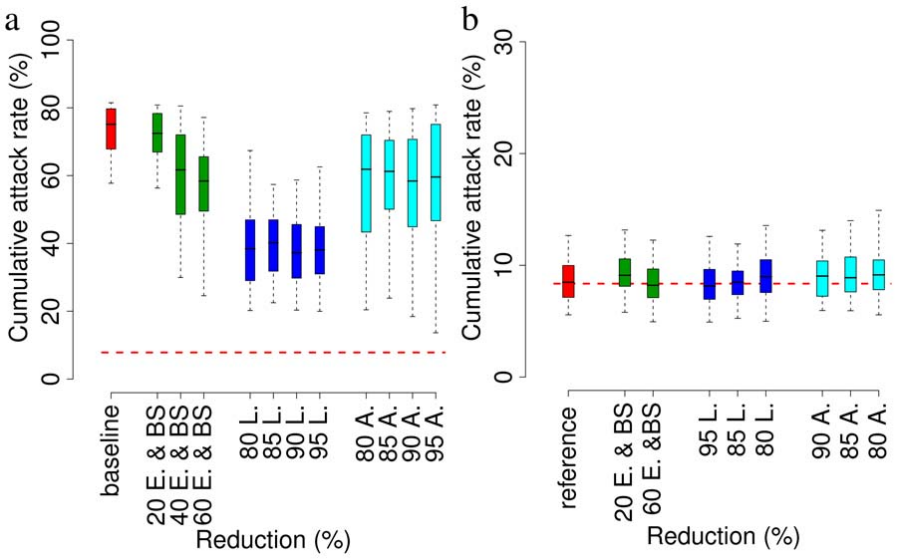
Sensitivity analysis. **a** Red: distribution (2.5%, 25%, 50%, 75% and 97.5% percentiles) of the cumulative attack rate (only symptomatic cases are considered) by assuming no interventions (baseline scenario) as in Fig. 5a and Fig. 5b, and 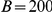 ha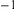. Green: as in the baseline scenario but for reductions of breeding sites and eggs. Blue: as in the baseline scenario but for reductions of larvae. Cyan: as in the baseline scenario but for reductions of adults. The horizontal dashed red line represent the observed attack rate (symptomatic cases, obtained by multiplying the observed prevalence, 10.2% [18], by the probability of developing clinical symptoms, 0.82 [18]). **b** Red: distribution (2.5%, 25%, 50%, 75% and 97.5% percentiles) of the cumulative attack rate (only symptomatic cases are considered) by assuming the same intervention as in Fig. 5c and Fig. 5d (reference scenario), namely reduction of 40% as for breeding sites and eggs, 90% as for larvae and 95% as for adults, and 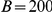 ha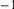. Green: as in the reference scenario but for different reductions of breeding sites and eggs. Blue: as in the reference scenario but for different reductions of larvae. Cyan: as in the reference scenario but for different reductions of adults. The horizontal dashed red line represents the observed percentage of symptomatic cases as resulting from survey data [18].

Let us now consider two aspects of the control strategy. Firstly, we assume different efficacy in terms of reduction of breeding sites, eggs, larvae and adults to evaluate the robustness of the estimated effects of the interventions undertaken in Italy. As shown in Fig. 6b, results are robust for small variations of the efficacy of the vectors control. In a fully susceptible population the time from primary index case to secondary infections was estimated to be 11 days on average (95% CI 3–20). This allows public health authorities to gain time to put in place control measures.

Secondarily, we investigate the efficacy of the single interventions (breeding sites removal, larvicides, adulticides). Results are shown only for 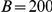 ha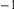. As shown in Fig. 6a, reduction of eggs and breeding sites could be effective only by hypothesizing a massive intervention (cumulative attack rate is reduced on average from 73% to 55% by reducing eggs and breeding sites of 60%); adulticides do not contribute much to reducing the overall number of cases (cumulative attack rate is reduced on average from 73% to 60%); larvicides contribute to a substantial reduction of the overall number of cases (cumulative attack rate is reduced on average from 73% to 40%); In fact, larvicides are effective for a prolonged period of time and thus can contribute to decrease systematically the number of adults for a long period of time and, consequently, to substantially reduce the overall attack rate. Quite the contrary, adulticides were used for a very limited period of time (3 days) and thus their effect is limited due to the rapid increase of adults suddenly after the intervention. Overall, these results suggest that only a combined intervention, as the one performed during the outbreak, can result in a drastic decrease of the number of cases.

Five smaller clusters of local transmission were detected in five towns in the same region (i.e., Ravenna, Cervia, Cesena, Rimini, and Bologna). Cervia and Ravenna are the main towns of the municipalities where the two most affected villages (Castiglione di Cervia and Castiglione di Ravenna) are located. The two affected villages account for the 2.1% of the population of the municipalities of Cervia (27,000 inhabitants) and Ravenna (157,000 inhabitants). By analyzing commuting data of the Emilia–Romagna region [47], we found that the number of individuals traveling daily to Cervia and Ravenna for work or study is 8,787 (from 249 different municipalities), and the number of persons traveling daily from Cervia and Ravenna to other municipalities is 10,861 (towards 139 different municipalities). The exact number of commuters for Castiglione di Cervia and Castiglione di Ravenna is unknown but it should not exceed 2.1% of the overall number of commuters. However, the probability of traveling from/to a certain municipality should be similar to that observed for the two municipalities as a whole and we found that clusters of local transmission were recorded in municipalities well connected with the municipalities of Ravenna and Cervia (see Table 4). For at least four of the five clusters, population movement (i.e., persons who visited the area that was primarily affected or persons from the primarily affected area who visited one of the four towns) can be reasonably assumed to have been the main determinant of local transmission. Another possible explanation is passive vector mobility (e.g. infected mosquitoes transported by car from the initial cluster), since the flight range (active mobility) is usually considered to be less than 1 km [35], [48], [49].

**Table 4:** Commuting patterns.

municipality	origin (rank)	destination (rank)	cases [63]
Cesena	18% (2)	10% (3)	15
Rimini	2.9% (10)	3.1% (10)	6
Bologna	9.5% (3)	1.6% (13)	5
Cervia	-	-	19
Ravenna	-	-	9

Probability of origin and destination (and rank over all possible origins/destinations) of individuals commuting daily for work or school from/to other municipalities were local clusters of transmission were observed. The two most affected villages are located in the municipalities of Cervia and Ravenna.

Our results suggest that the transmission potential of CHIKV in Italy was similar to the one observed in tropical regions where Chikungunya fever is widespread (e.g., Reunion Island, where the best estimate for the initial 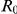 was 3.7 [3]). Specifically, we estimated 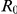 to be in the range of 1.8–6. However, being the reproduction number strongly dependent on the density of mosquitoes, which in turn varies a lot over time as a consequence of seasonal meteorological effects, different (even larger) values of 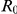 could be observed in future outbreaks, depending on the time of epidemic seeding. In [3], by adapting a method originally introduced in [50] for human–to–human infections, 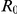 was estimated from the generation interval probability distribution function and the number of gonotrophic cycles of the mosquito. This method can not be applied in our study, as the undertaken control measures have contributed to alter the gonotrophic cycles of the mosquito in a indeterminable manner. We found that the probability of observing a major outbreak after the introduction of an index case depends on the ratio of mosquitoes to humans and was estimated to be in the range of 32%–76%. These results confirm the high risk to Europe of tropical vector–borne diseases as a consequence of globalization, which has been modifying the mobility of humans and vectors. Climate changes could have been playing a role, as the geographical limits of mosquito–borne diseases can be influenced by climate [51], [52], but this is still debated [53]–[55].

Moreover, our analysis strongly support the efficacy of the disinfestation strategy performed during the Italian outbreak, which drastically contributed to reduce the cumulative attack rate (of about 88%), though the application of self–protection preventive measures (insect repellents and window screens) could also have played a role [18]. Therefore, even if the transmission potential of Chikungunya virus could be sensibly high also in temperate climate countries, the epidemic can be controlled by performing timely interventions.

The proposed model has several limitations. We assume exponential distribution for all parameters of model 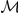 related to the length of the different periods (e.g. latency, infectiousness, etc.), though it would be preferable to use multiple classes within each group to give more realistic gamma distributed lifetimes (see for instance [56]). We assume density–dependent growth only in eggs, though other density–dependent regulating processes should be considered for other lifestages of the mosquitoes, e.g. larvae and pupae [25]. These modeling choices are due to the lack of data for parametrizing the model. The lack of information on the actual number of breeding sites – it could be assessed only by performing a field study – prevent us to give precise estimates on the density of mosquitoes over time in the study area. However, we would note that our estimates of 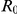, attack rates and probability of major outbreak are robust with respect to assumptions on the number of breeding sites. Moreover, the temporal dynamics of the vector is qualitatively well captured by model 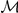 (though not in terms of absolute abundance) and this allowed us to clarify whether or not the sharp decrease in the number of cases observed after the intervention was due to the intervention itself or to the spontaneous reduction of adults due to decrease of temperature. Definitely, a key lesson learnt from the analysis of the Chikungunya outbreak in Italy is the necessity to improve tools for obtaining reliable, though costly, estimates of the vector density (see for instance [57]) – thus bypassing the necessity of developing ad-hoc models. The proposed model, describing the temporal dynamics of *Aedes albopictus*, provides a valid alternative in the absence of reliable field data.

## Supporting Information

Figure S1
**Parameters optimization.** **a** Green points represent the values of the score function 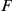 plotted versus the number of notified cases as predicted by the model (with 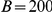 ha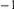) before intervention for 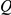 different values of the model parameters as obtained by the LHS procedure. Red point represents the minimum of 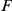. Black points represent the values of the score function 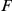 plotted versus the number of notified cases as predicted by the model before intervention as obtained by repeating 100 times the optimization procedure. The inset shows the minimum of 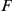 for the 100 replicates (red points). The blue vertical line represent the number of notified cases reported to the surveillance system before intervention, namely 132. **b** As **a** but for 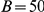 ha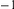.(TIF)

Figure S2
**Results for increasing number of simulations.** **a** Mean (red points), median (blue points) and 95% CI (shaded grey area) of 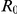 for increasing number of simulations with 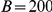 ha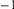 in the absence of interventions (baseline scenario). **b** As **a** but for probability of observing a major outbreak. **c** As **a** but for the cumulative number of symptomatic cases. **d** As **a** but for the biting rate.(TIF)

Figure S3
**Temporal dynamics of the mosquito.** **a** Average density (number per ha) of adult female mosquitoes over time as predicted by the model by assuming 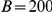 ha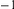 (green line) and 95% CI (grey area). **b** As **a** but for 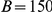 ha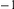. **c** As **a** but for 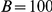 ha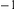. **d** As **a** but for 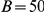 ha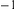.(TIF)

Figure S4 **Range of the optimal parameters values.** Distribution of the model parameters (2.5%, 25%, 50%, 75% and 97.5% percentiles) after LHS optimization. Numbers below and over the boxplot represent the explored range of values.
(TIF)

Figure S5
**Epidemic threshold and probability of major outbreak.** **a** Epidemic threshold 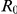 in relation to biting rate 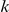 and ratio of mosquitoes to humans 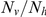. The black line represents the average threshold condition and the shaded blue area represents 95% CI, as resulting from uncertainty of model parameters. The red rectangle identifies the likely range of the two parameters in the two Italian villages affected by CHIKV. **b** Probability of observing a major outbreak as a function of the ratio of mosquitoes to humans 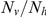 for two extreme values of the biting rate 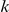, namely 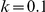 days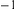 in red (solid line black represents the average probability and the shaded area represents 95% CI) and 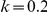 days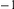 in blue.(TIF)

Figure S6
**Baseline simulations and reference interventions.** **a** Average daily number of symptomatic notified cases as predicted by the model in the absence of interventions (baseline scenario, blue line, scale on the left) and 95% CI (grey area) by assuming 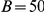 ha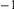, compared to the actual daily number of symptomatic notified cases (black points). Red line represents the overall average daily number of symptomatic cases as predicted by the model. Green line represents the average density of mosquitoes (scale on the right). **b** Histogram of the cumulative number of symptomatic cases as predicted by the model in the absence of interventions. **c** and **d** As **a** and **b** respectively but for assuming an intervention resulting in the following reductions: 40% as for breeding sites and eggs, 90% as for larvae and 95% as for adults (reference scenario).(TIF)
