# Supplementary figures and images for: Transmission Potential of Chikungunya Virus and Control Measures: The Case of Italy
Source: PLoS One. 2011 May 3;6(5):e18860. doi: 10.1371/journal.pone.0018860 (PMC3086881; doi:10.1371/journal.pone.0018860)

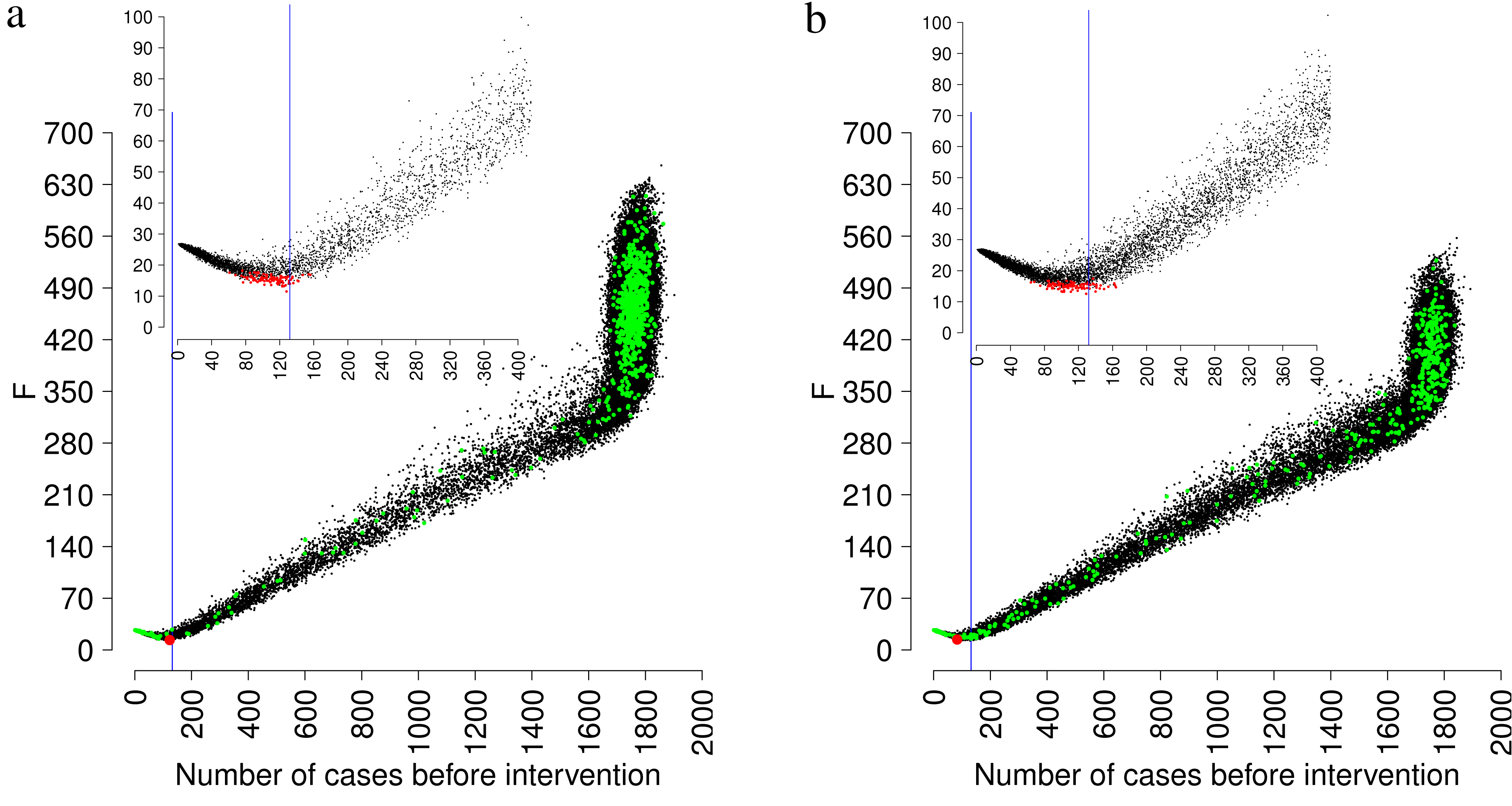

Supplement: Figure S1 — Parameters optimization. a Green points represent the values of the score function plotted versus the number of notified cases as predicted by the model (with ha) before intervention for different values of the model parameters as obtained by the LHS procedure. Red point represents the minimum of . Black points represent the values of the score function plotted versus the number of notified cases as predicted by the model before intervention as obtained by repeating 100 times the optimization procedure. The inset shows the minimum of for the 100 replicates (red points). The blue vertical line represent the number of notified cases reported to the surveillance system before intervention, namely 132. b As a but for ha. (TIF) [file pone.0018860.s001.tif]

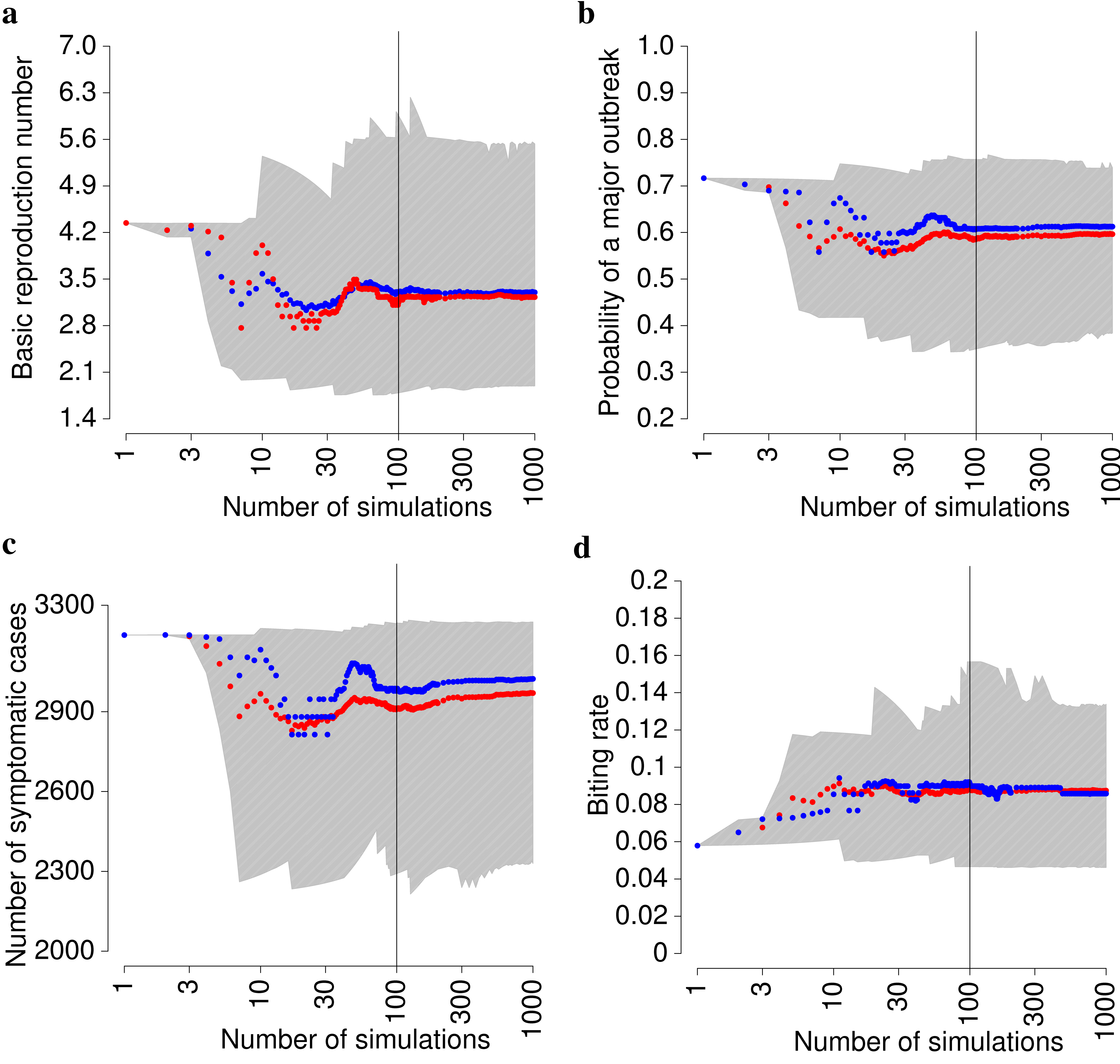

Supplement: Figure S2 — Results for increasing number of simulations. a Mean (red points), median (blue points) and 95% CI (shaded grey area) of for increasing number of simulations with ha in the absence of interventions (baseline scenario). b As a but for probability of observing a major outbreak. c As a but for the cumulative number of symptomatic cases. d As a but for the biting rate. (TIF) [file pone.0018860.s002.tif]

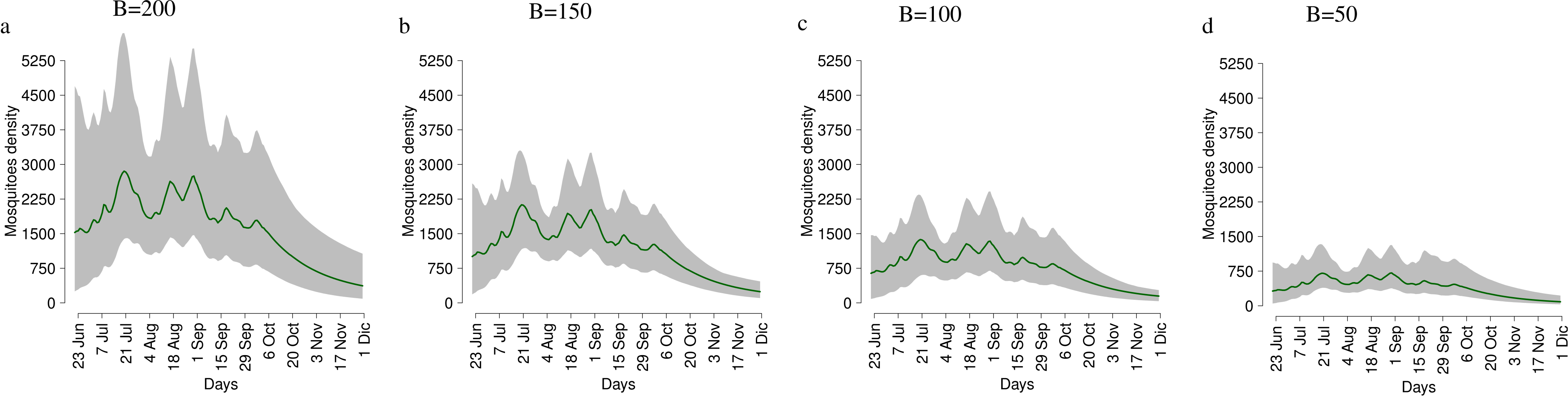

Supplement: Figure S3 — Temporal dynamics of the mosquito. a Average density (number per ha) of adult female mosquitoes over time as predicted by the model by assuming ha (green line) and 95% CI (grey area). b As a but for ha. c As a but for ha. d As a but for ha. (TIF) [file pone.0018860.s003.tif]

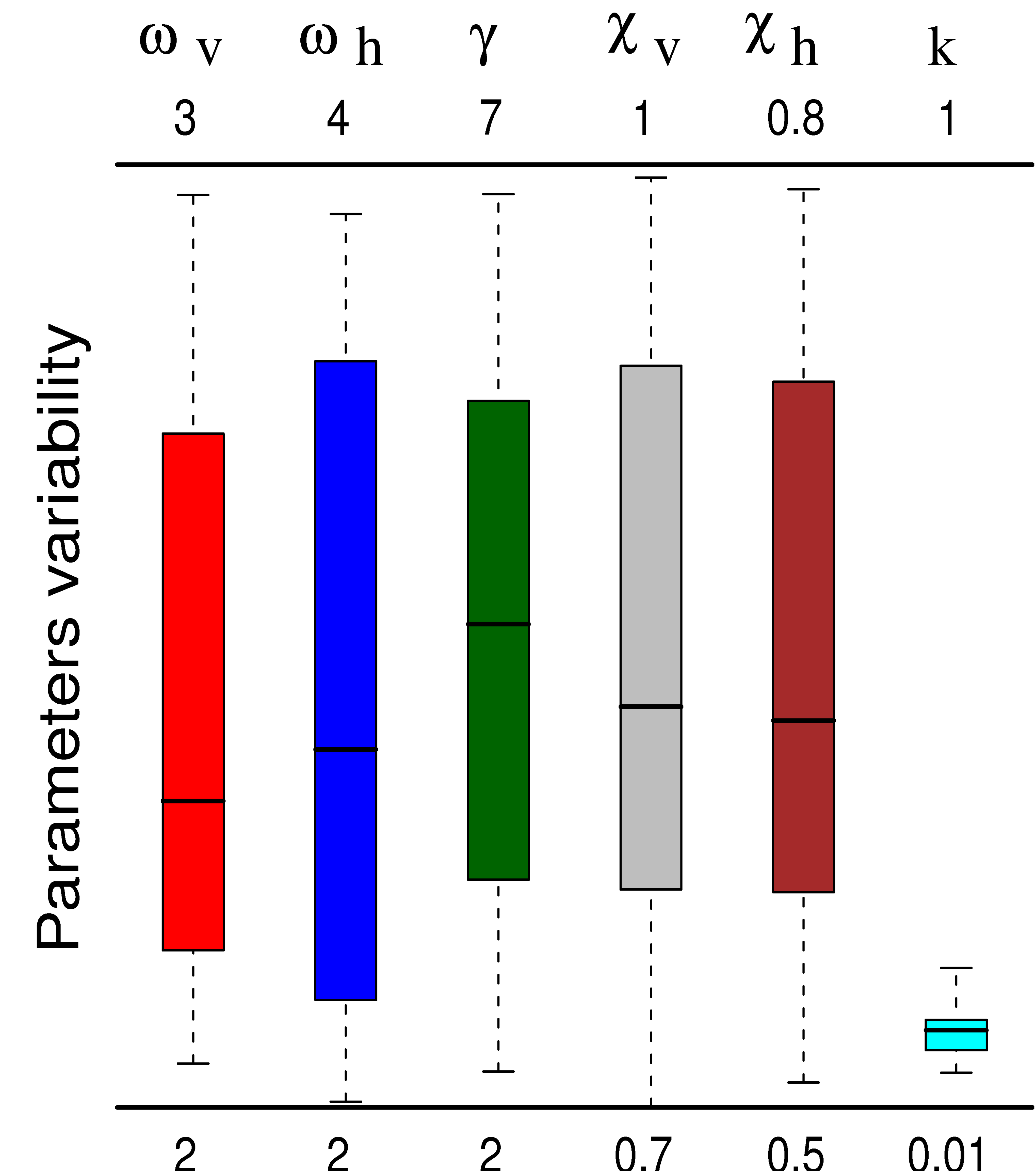

Supplement: Figure S4 — Range of the optimal parameters values. Distribution of the model parameters (2.5%, 25%, 50%, 75% and 97.5% percentiles) after LHS optimization. Numbers below and over the boxplot represent the explored range of values. (TIF) [file pone.0018860.s004.tif]

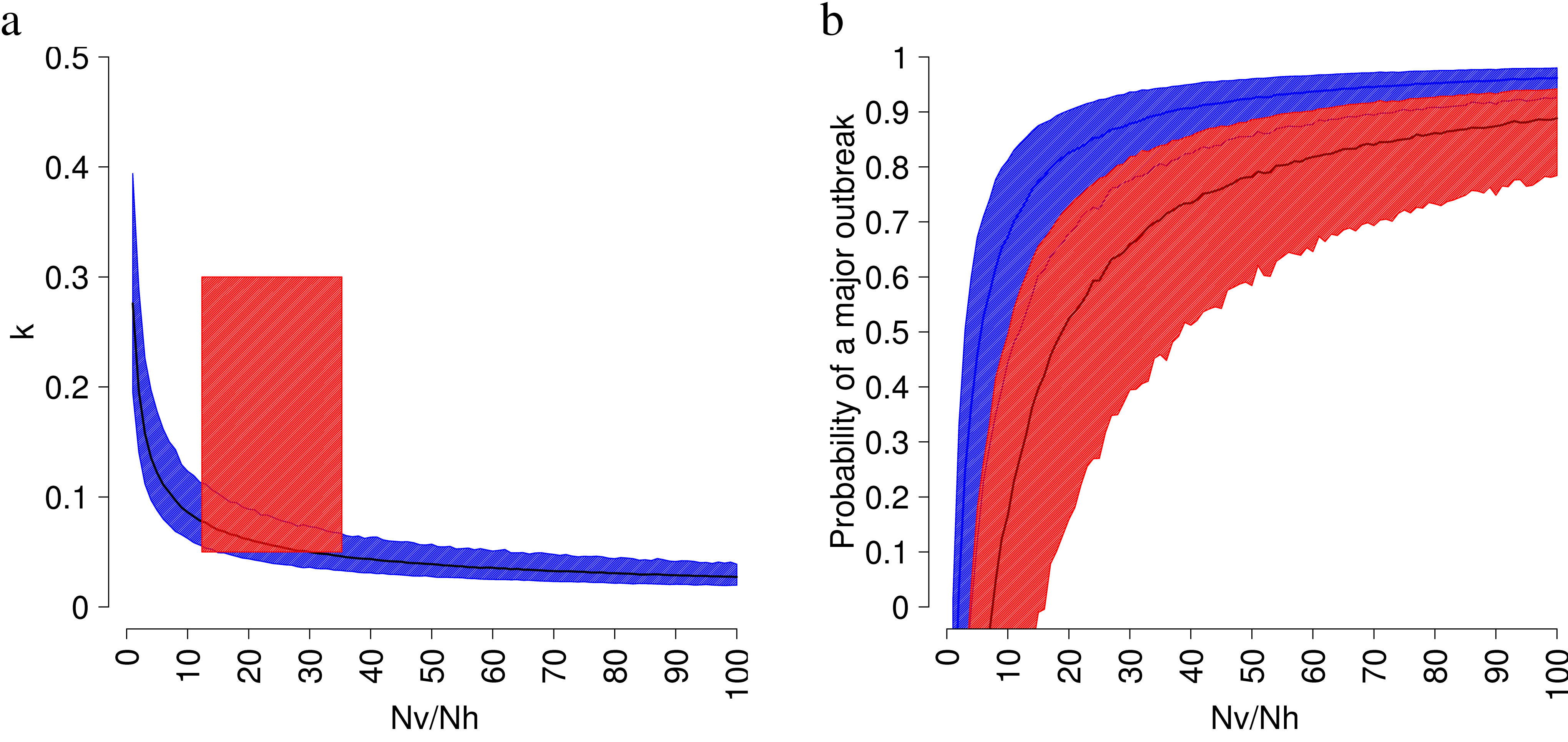

Supplement: Figure S5 — Epidemic threshold and probability of major outbreak. a Epidemic threshold in relation to biting rate and ratio of mosquitoes to humans . The black line represents the average threshold condition and the shaded blue area represents 95% CI, as resulting from uncertainty of model parameters. The red rectangle identifies the likely range of the two parameters in the two Italian villages affected by CHIKV. b Probability of observing a major outbreak as a function of the ratio of mosquitoes to humans for two extreme values of the biting rate , namely days in red (solid line black represents the average probability and the shaded area represents 95% CI) and days in blue. (TIF) [file pone.0018860.s005.tif]

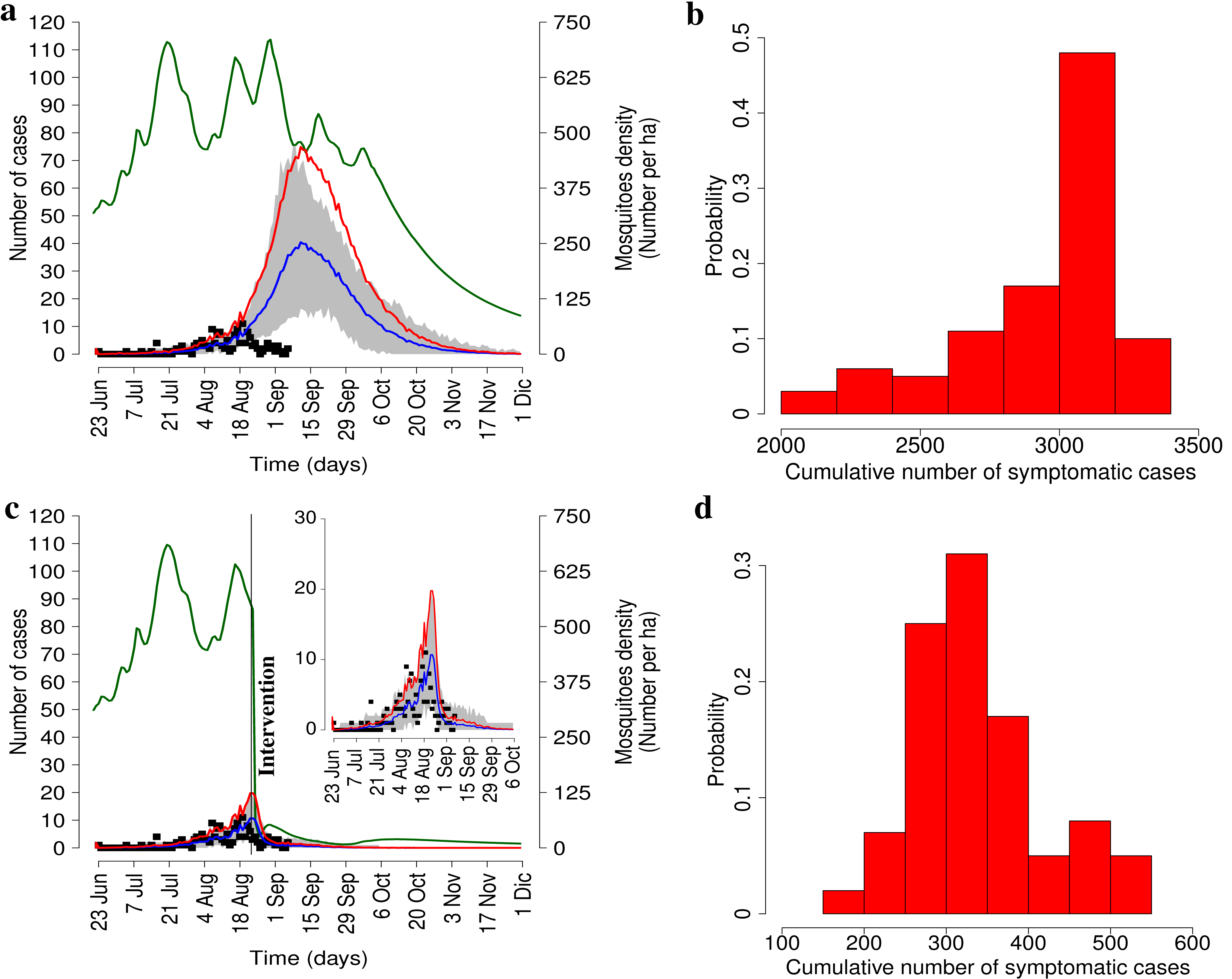

Supplement: Figure S6 — Baseline simulations and reference interventions. a Average daily number of symptomatic notified cases as predicted by the model in the absence of interventions (baseline scenario, blue line, scale on the left) and 95% CI (grey area) by assuming ha, compared to the actual daily number of symptomatic notified cases (black points). Red line represents the overall average daily number of symptomatic cases as predicted by the model. Green line represents the average density of mosquitoes (scale on the right). b Histogram of the cumulative number of symptomatic cases as predicted by the model in the absence of interventions. c and d As a and b respectively but for assuming an intervention resulting in the following reductions: 40% as for breeding sites and eggs, 90% as for larvae and 95% as for adults (reference scenario). (TIF) [file pone.0018860.s006.tif]
